# Светлой памяти Майорова Александра Юрьевича

**DOI:** 10.14341/probl13227

**Published:** 2022-12-20

## Abstract

1 января 2023 года на 59-м году жизни скоропостижно скончался заведующий отделом прогнозирования и инноваций диабета Института диабета ГНЦ-ФГБУ «НМИЦ эндокринологии» Минздрава России, президент Общероссийской Общественной организации инвалидов «Российская диабетическая ассоциация», врач-эндокринолог и диабетолог, доктор медицинских наук, профессор Майоров Александр Юрьевич.

## Abstract (en)

On January 1, 2023, at the age of 59, the head of the Diabetes Prediction and Innovation Department of the Diabetes Institute of the State Scientific Center-FGBU «NMITs of Endocrinology» of the Ministry of Health of Russia, the president of the All-Russian Public Organization of the Disabled «Russian Diabetes Association» endocrinologist and diabetologist, doctor of medical sciences, died suddenly Professor Mayorov Alexander Yurievich.

1 января 2023 года наш коллектив безвременно покинул Майоров Александр Юрьевич. Мы потеряли замечательного друга и коллегу, талантливого врача-эндокринолога и диабетолога, прекрасного ученого, выдающегося общественного деятеля. Заведующий отделом прогнозирования и инноваций диабета Института диабета ГНЦ-ФГБУ «НМИЦ эндокринологии» Минздрава России, президент Общероссийской Общественной организации инвалидов «Российская диабетическая ассоциация», доктор медицинских наук, профессор Майоров Александр Юрьевич (16 ноября 1964 г. – 01 января 2023 г.) скоропостижно скончался на 59-м году жизни.

Александр Юрьевич прожил яркую и профессионально насыщенную жизнь, всецело посвященную беззаветному служению медицинской науке и пациентам.

После окончания в 1987 г. с отличием Московского медицинского стоматологического института А.Ю. Майоров был зачислен в клиническую ординатуру Всесоюзного эндокринологического центра Академии медицинских наук СССР (ныне — ГНЦ-ФГБУ «НМИЦ эндокринологии» Минздрава России), и с тех пор его трудовая деятельность неразрывно связана с этим центром. За ординатурой последовала очная аспирантура, защищены кандидатская и докторская диссертации. В 2009 г. А.Ю. Майоров возглавил отделение психосоциальной реабилитации и обучения больных сахарным диабетом, а с 2017 г. — отдел прогнозирования и инноваций диабета. В этом же году стал профессором кафедры «Диабетология и диетология» Института высшего и дополнительного профессионального образования «НМИЦ эндокринологии» Минздрава России. А.Ю. Майоров был не только прекрасным врачом, но и прирожденным педагогом. Недаром с его именем связывают важнейшую всероссийскую программу «терапевтического обучения» пациентов с сахарным диабетом, которая стартовала в 1989 г. в рамках Федеральной целевой программы «Сахарный диабет», а с 2023 г. включена в Федеральный проект «Борьба с сахарным диабетом».

В 2019 г. А.Ю. Майоров вместе с коллегами стал лауреатом премии «Призвание» среди лучших врачей России «за создание нового направления в медицине — “Школы для больных диабетом”». Как глубокий и неординарный ученый, А.Ю. Майоров внес весомый вклад в изучение вопросов патогенеза сахарного диабета 2 типа. Ему удалось внедрить в России сложную технологию оценки чувствительности тканей к инсулину (клэмп-метод), разработать методики оценки биоэквивалентности и фармакокинетики новых препаратов инсулина, стать лидером в испытании новых лекарственных препаратов в области диабетологии. На счету профессора Майорова А.Ю. более 240 научных публикаций, разнообразные структурированные программы обучения больных сахарным диабетом, десятки монографий, национальных клинических рекомендаций по диагностике и лечению сахарного диабета.

За самоотверженную и результативную профессиональную деятельность А.Ю. Майорову в составе коллектива в 2012 г. присвоено звание Лауреата Премии Правительства РФ за «создание и внедрение в практику здравоохранения Российской Федерации системы современных технологий диагностики, лечения и профилактики сахарного диабета».

Александр Юрьевич известен не только в нашей стране, но и за рубежом как лидер общественной пациентской организации «Российская диабетическая ассоциация» (РДА). С 2014 г. А.Ю. Майоров как руководитель РДА успешно отстаивал права и интересы пациентов с сахарным диабетом, представлял нашу страну во многих международных медицинских организациях, в том числе в составе правления Европейского региона Международной Диабетической Федерации (IDF Europe).

Руководство и коллектив «НМИЦ эндокринологии» Минздрава России, пациентское и экспертное сообщества выражают глубокие соболезнования по случаю тяжелой и невосполнимой утраты.

Память о докторе Александре Юрьевиче Майорове навсегда останется в наших сердцах, а также в сердцах миллионов граждан России, — настолько важной и значимой для многих была кипучая и чрезвычайно эффективная врачебная и научно-исследовательская деятельность этого необыкновенно умного, невероятно доброго и отзывчивого, душевного и прекрасного человека, отдавшего всего себя без остатка своим пациентам, служению во имя и на благо Отечества.

Редакция и читатели журнала «Проблемы эндокринологии»искренне приносят соболезнования родным, близким, коллегам и ученикам А.Ю. Майорова.

